# Trends in Atopic Dermatitis—From Standard Pharmacotherapy to Novel Drug Delivery Systems

**DOI:** 10.3390/ijms20225659

**Published:** 2019-11-12

**Authors:** Eliana B. Souto, João Dias-Ferreira, Jéssica Oliveira, Elena Sanchez-Lopez, Ana Lopez-Machado, Marta Espina, Maria L. Garcia, Selma B. Souto, Carlos Martins-Gomes, Amélia M. Silva

**Affiliations:** 1 Department of Pharmaceutical Technology, Faculty of Pharmacy, University of Coimbra (FFUC), Pólo das Ciências da Saúde, 3000-548 Coimbra, Portugal; j.dias.ferreira@outlook.pt (J.D.-F.); j.alexandra.3@hotmail.com (J.O.); esanchezlopez@ub.edu (E.S.-L.); lora_ana@hotmail.com (A.L.-M.); 2 CEB—Centre of Biological Engineering, University of Minho, Campus de Gualtar 4710-057 Braga, Portugal; 3 Department of Pharmacy and Pharmaceutical Technology and Physical Chemistry, Faculty of Pharmacy, University of Barcelona, Ave. Joan XXIII, 08028 Barcelona, Spain; m.espina@ub.edu (M.E.); marisagarcia@ub.edu (M.L.G.); 4 Institute of Nanoscience and Nanotechnology (IN2UB), University of Barcelona, Barcelona 08028, Spain; 5 Department of Endocrinology, Hospital de São João, Alameda Prof. Hernâni Monteiro, 4200-319 Porto, Portugal; sbsouto.md@gmail.com; 6 Centre for Research and Technology of Agro-Environmental and Biological Sciences (CITAB), University of Trás-os-Montes e Alto Douro (UTAD), Quinta de Prados, 5001-801 Vila Real, Portugal; gomes.ma.carlos@gmail.com (C.M.-G.); amsilva@utad.pt (A.M.S.); 7 Department of Biology and Environment, University of Trás-os-Montes e Alto Douro (UTAD), Quinta de Prados, 5001-801 Vila Real, Portugal

**Keywords:** atopic dermatitis, drug delivery systems, pharmacological treatment, nanotechnology, nanoparticles

## Abstract

Atopic dermatitis (AD) is a predominant and deteriorating chronic inflammation of the skin, categorized by robust burning and eczematous lacerations in diverse portions of the body. AD affects about 20% of both offspring and adults worldwide. The pathophysiology of AD combines environmental, hereditary, and immunological aspects, together with skin barrier dysfunction. The procedures used to prevent the disease are the everyday usage of creams to support the restoration of the epidermal barrier. The classical treatments include the use of topical corticosteroids as a first-line therapy, but also calcineurin inhibitors, antihistamines, antibiotics, phototherapy, and also immunosuppressant drugs in severe cases of AD. Topical drug delivery to deeper skin layers is a difficult task due to the skin anatomic barrier, which limits deeper penetration of drugs. Groundbreaking drug delivery systems, based on nanoparticles (NPs), have received much attention due to their ability to improve solubility, bioavailability, diffusion, targeting to specific types of cells, and limiting the secondary effects of the drugs employed in the treatment of AD. Even so, additional studies are still required to recognize the toxicological characteristics and long-term safety of NPs. This review discusses the current classical pharmacotherapy of AD against new nanoparticle skin delivery systems and their toxicologic risks.

## 1. Introduction

Atopic dermatitis (AD) is a chronic skin illness characterized by itchy, reddish, and scaly lesions along with a continuous inflammatory pattern [1]. AD arises commonly in familial context with a prevalence of atopic conditions, as allergic rhinitis or bronchial asthma, and food allergies [2]. Topical administration of drugs is still the main therapeutic approach for AD. Despite this, several disadvantages may be cited as reduced patient compliance (a consequence of adverse effects as skin irritation or allergy), low efficiency, and specificity of these systems in delivering therapeutic drugs [3]. Innovative drug delivery systems should, therefore, exhibit the capacity to penetrate the *stratum corneum* (SC), which is naturally impermeable to a range of substances, in order to reduce adverse effects and increase drug targeting [3,4,5]. There is still no medication capable of reversing the pathological effects of AD. However, formulations based on nanoparticles (NPs) have been exploited for topical administration of drugs and are expected to overcome the above-mentioned limitations [6,7,8].

## 2. Pathophysiology

AD, also called atopic eczema, is the most common skin disease, characterized by a pattern of pruritus, skin deterioration, and chronic inflammation [1,6,7]. This illness affects individuals in early-, mid- or late-stages of their lives and does not have a known cure so far. Over the course of the disease, the levels of IgE increase drastically leading to cutaneous signals that appear in early ages and persist until late stages of life, when they begin to disappear. Because of this, AD is also labelled as “allergic march” [2].

AD results from a multiplicity of factors such as environmental injuries, impairments against the natural barrier of the skin and response of the immune system. This latter enhances the activity of mast cells and T-lymphocytes, followed by higher levels of IgE in patients serum, inflammatory signals, and pruritus [9,10,11]. Very common features amongst AD patients are pruritus, papules related to erythematous pruritic lesions, xerosis, skin rashes, and serous exudate as the result of the persistent inflammation [2,12]. The eyelid often presents a second fold in the skin, known as Dennie-Morgan fold [9,11,12]. Two theories are advanced to clarify the skin disruption and appearance of cutaneous lesions/skin rash. The first, named “in-out”, is based on an imperfect skin barrier. The second, called “inside-out”, originated from the concept of the adaptive immune system burden. The first theory is more recent, and its foundations concern the vital role of skin impairment in the immune system activation. The two theories are not self-excluded, but are complementary [13,14,15].

### 2.1. Skin Barrier

The normal function of the skin is regulated primarily by both physical and chemical integrity of this barrier, limiting both the loss of water, and the entrance of pathogens and immunogenic molecules. In this way, it avoids the contact of such molecules with antigen-presenting cells, and thereby, limits the response of the immune system [11,16]. When the skin barrier functions fail, the risk of proliferation of microorganisms and of invasion of antigens increases [2,15].

The mechanisms allowing the disruption of this barrier are based on three main changes in skin homeostasis, namely, (i) alterations in lipid composition and secretion, (ii) fluctuations in the differentiation processes of the epidermal terminal, and (iii) mutations in the gene coding filaggrin (filament-aggregating protein), due to its role in the synthesis of natural hydration factor (which upholds keratinocytes linked) and in the differentiation of epidermal terminal. Missense or nonsense mutations lead to altered or even a loss of function of the gene. In the case of mutations in the filaggrin gene, these are related to the enhanced risk of developing AD. These effects are the result of higher skin pH, triggering phenomena, such as serine proteases, increased synthesis of cytokines, and the development of *Staphylococcus aureus* (*S. aureus*) with parallel colonization of microbes. The higher the density of these pathogens in the skin, the higher the seriousness in the clinical setting of AD [11,14,16].

Skin homeostasis is regulated by a myriad of factors that govern the capacity of skin cells to divide, proliferate, and differentiate. Sphingolipids (SPL) are a class of molecules that interfere in the processes of cell regulation with functions at the structural and biological levels [17]. AD is currently associated with SPL and ceramides (CR) impairment at the epidermal level (namely in the SC), when compared to the skin of aged patients. The lack of SPL and CR is a key factor in the development of dehydrated and disrupted skin. The physiological concentration of ceramides in the SC is regulated by three vital enzymes, β-glucocerebrosidase, sphingomyelinase, and ceramidase, whose function is to hydrolyze the former [18]. In the case of AD patients, a de novo enzyme was identified, named sphingomyelin/glucosylceramide deacetylase, which catalyzes the hydrolysis of glucosylceramide or sphingomyelin at the level of the acyl group. In these patients, it has an activity increased five-fold when compared to normal individuals [19]. This massive increase explains the reduced levels of both CR and sphingosine. The decrease of these two molecules is also connected to a reduced transepidermal water loss and the faint levels of sphingosine are related to the risk of bacterial infections. Studies on skin dysfunction in AD demonstrated the loss in the synthesis of ceramides, which is essential for healthy skin. The biosynthesis of ceramides differs among ethnic groups, and there is still no connection between the filaggrin gene and ceramide synthesis [19,20]. A study, performed using SPL extracts, demonstrated the potential positive impact in the production of ceramides, which leads to an ordered SC and an improvement in AD status [21]. Another study, this time using a cream composed of ceramides and magnesium, demonstrated higher benefits in the skin over other methods, such as corticosteroids (which modify skin moisturizing factors) or even lipid-enriched emollients [22]. Current efforts are being driven to scrutinize the pathophysiology of AD and to yield molecular targets for future pharmacological treatments.

### 2.2. Skin Immunology

Skin immunology suggests the existence of a discrepancy amongst the action of innate and adaptive immune systems, triggering an irregular reply of T-cells. The result is an increase in the synthesis of interleukins (IL-4, IL-5, IL-13), which hinder the differentiation of the Th1-cells, as well as increase the synthesis of IgE, which elevate the levels present in about half of the occurrences [8,12].

## 3. Epidemiology

Epidemiological data point to about 20% of probability of an individual to develop AD during its lifetime. The prevalence of the disease is about 15–30% both, in children and in adults [23]. While, the total prevalence of AD depends on the country/region, a marked increase has been noted between 1950 and 2000, a phenomenon termed “allergic epidemy” [12], with the highest incidence in industrialised countries [23]. AD develops in 95% of the cases in children aged under five-years-old [12], but overall, 60% of the AD cases are reported in children under one-year old [11]. Nevertheless, AD likewise disturbs adults, accounting for 1% to 3% of prevalence [24].

## 4. Clinical Diagnosis

AD is currently diagnosed via clinical observation. This procedure is still needed due to the lack of specific and validated laboratory assays to rapidly determine the presence or absence of the illness [25]. When clinical observations are questionable, skin biopsies are often performed to clear-cut other pathologies, as T-cell lymphoma, which trigger the same signals and symptoms [26,27]. Briefly, the combination of an exact anamnesis and an appropriate physical examination are crucial for an improved diagnosis. Physicians should be focused on the distribution and morphology of the lesions and relate them to the age of the patient [2]. Other features that are considered critical in the diagnostic process are the presence of ichthyosis vulgaris, pruritus, xerosis, and relapsing eczematous lesions [9]. The injuries identified according to the symptomatology are: Allergic contact eczema, irritant eczema, toxic contact eczema, infective eczema, and nummular eczema. Middle-aged patients show symptoms that may resemble AD as eczematous rashes derived from pathological conditions, such as scabies, erythroderma, psoriasis, seborrheic dermatitis, cutaneous T-cell lymphoma, and immune deficiency syndromes. In such circumstances, a differential set of diagnostic tests is mandatory [28].

## 5. Prevention

To avoid AD three strategies are considered. The first relates to the exclusion of food-derived allergens. The second concerns the reduction, as much as possible, of the contact with antigen sources from pollens, mite dust, traffic or tobacco smoke, volatile organic compounds, and animal fur [29,30,31]. The third combines these two strategies plus the employment of daily skin emollients and lotions [2,31,32]. So far, it has been noted that using skin emollients on daily regimens decreases the risk of children developing AD by 30% to 50% [33,34].

## 6. Non-Pharmacological and Pharmacological Treatments

AD treatments have four main aims, namely; improving patients’ quality of life, reducing the severity of the pathology, preventing further infections and controlling the illness in the long-term [11,35]. Table 1 provides an overview of the current therapeutic approaches in AD. Presently used therapeutic approaches recommend skin hydration, skin inflammation control, and renewal of skin structure by application of emollients [6,7,26]. The use of moisturizers to soften AD-diseased skin is one of the principal treatment approaches. Pharmacological approaches consist of the use of topical corticosteroids to reduce inflammation, antibiotics (namely antimicrobials) to eradicate infections induced by bacteria or other parasites, antihistamines to reduce pruritic symptoms, and calcineurin inhibitors to prevent eczema propagation and to reduce inflammation as well. Phototherapy and immunomodulation by systemic administration of immunosuppressant drugs are also proposed [25,36].

### 6.1. Non-Pharmacological Approaches

#### 6.1.1. Moisturizers

The composition of a skin moisturizer is complex and consists of using emollients, humectants, and occlusive agents. Commonly used Humectants, include glycerol, urea, or lactic acid; amongst the most frequent included emollients are glycol stearate, glyceryl stearate, and soy-derived sterols; while the occlusive agents include petrolatum, dimethicone, and mineral oils [28], as well as nanoparticles [37]. The choice of the emollient depends on the distinct characteristics of the skin of each patient. Emollients applied topically are commonly employed in dry and lichenified skin, and should ensure extensive skin lubrication [35]. To prevent skin water loss, occlusive agents are used due to their ability to generate an occlusive coating. Humectants also improve the hydration of skin but, instead of generating an occlusive layer, they retain the water molecules via penetration through the skin (oppositely to occlusive agents that do not permeate across the skin) and promote SC hydration [10]. Daily moisturizers should not have scents or perfumes in their composition as these are potential allergens that can cause further dysfunction of the already diseased skin. Despite the currently available pool of data from the literature, there is still no evidence that emollients, composed of ceramides and fatty acids, are better than old-style emollients [25,35]. Emollients should, therefore, be considered in the primary care of AD [38].

#### 6.1.2. Bath and Wet Wraps

A daily bath is a mandatory habit in the life of an AD patient. It is intended to remove allergens, skin scales, and irritant substances accumulated every day on the surface of the skin [32]. The use of wat wraps reduces skin water loss, avoiding dry skin and further cutaneous rash and itching. The procedure should be performed as follows: Firstly, the patient must take a bath and, afterwards, apply corticosteroids or emollients directly on the skin; the treated skin regions are then wrapped using wet bandages and, only after that, the normal dry outfit. Topical application of corticosteroids has increased benefits when simultaneously used of wet wraps due to increased drug absorption [11,32].

### 6.2. Pharmacological Approaches

#### 6.2.1. Topical Corticosteroids

The primary treatment of AD includes topical corticosteroids. In children, this class of molecules must be carefully chosen, and only low-potency corticosteroids should be used. An exception to this rule applies only when low-potency corticosteroids do not present effective results. High-potency corticosteroids are, therefore, employed to avoid severe scenarios [35]. The management of this class of drugs must be understood by users and caretakers in order to minimize adverse effects (e.g., skin receding or skin stretch marks), which may result in low patients’ compliance due to corticophobia [25,33]. The physician’s recommendations and dose tunings are vital to expanding the success of corticotherapy [11].

#### 6.2.2. Antibiotic Treatments

Antibiotics are recommended in patients infected by *S. aureus* to prevent spreading of the microorganisms which, otherwise, infects other patients. Administration can be oral or intravenous. In cases of severe AD, the patients infected with *S. aureus* may undergo antibiotic therapy, aiding in the withdrawal of bacterial clusters and, thus, improving the clinical state of the patients. Despite this, the topical application of antibiotics is not yet proven to be advantageous in the treatment of concomitant infection with *S. aureus* in AD patients [32,35]. Nevertheless, the choice of the antimicrobial drug is of topmost importance in circumventing the resistance effects of the microorganisms [11].

### 6.3. Antihistamines

Pruritus is the most influential symptom of the disease affecting the life quality of these patients [11]. Antihistamines are commonly used to decrease the overall itching. However, these drugs are not used for the effective treatment of eczema, but for ameliorating the symptoms [25]. Scientific data are somehow contradictory, i.e., some describe no superiority of antihistamines when compared to placebo. Whereas, others show an antipruritic outcome [39,40]. An adverse effect of first-generation antihistamines is related to their capacity to sedate, which can be profitably used in AD patients that have a frequent disturbance of sleep [39,40].

#### 6.3.1. Calcineurin Inhibitors

Calcineurin inhibitors are classified as anti-inflammatory agents of non-steroidal origin. They exhibit their actions through blockage of activated T-cells cytokine transcription, leading to a decline in the inflammation degree [41]. The application of these drugs in the affected areas of the skin improves the condition without triggering skin shrivelling [35]. Calcineurin inhibitors are a second-line choice in short-term treatments. The simultaneous use of emollients and calcineurin inhibitors is strongly recommended. Nevertheless, the formulations must be applied at distinct time points to prevent a dilution result. Other cases of success are the topical use of immuno-modulators, namely pimecrolimus as a cream with mild-corticosteroid properties, and tacrolimus as a cream with moderate- to strong-corticosteroids properties. Both cases were shown to decrease excoriations, pruritus, and erythema [42].

#### 6.3.2. Phototherapy

The incidence of ultraviolet radiations—ultraviolet A (UVA) and ultraviolet B (UVB) - leads to cellular damages. Studies performed with UVB demonstrated its potential to reduce bacteria onto the skin, as well as the synthesis of superantigen by *S. aureus* [43]. A therapeutic approach for AD-derived eczema might consist of applying UVA and UVB together with topical corticosteroids. Despite this, the UVA and UVB radiations contribute to accelerating skin ageing and increased risk of skin cancer [44,45].

#### 6.3.3. Systemic Immunosuppressant Drugs

In life-threatening cases of AD, as when classical treatments like the topical administration of corticosteroids and phototherapy have lost their efficacy, methotrexate, mycophenolate mofetil, and azathioprine substances, belonging to the class of systemic immunosuppressant drugs, are the alternative approach [35]. Emollients must be used in parallel to re-establish the water-lipid film of the skin [11].

## 7. Nanotechnology for Topical Applications

**Figure 1:**
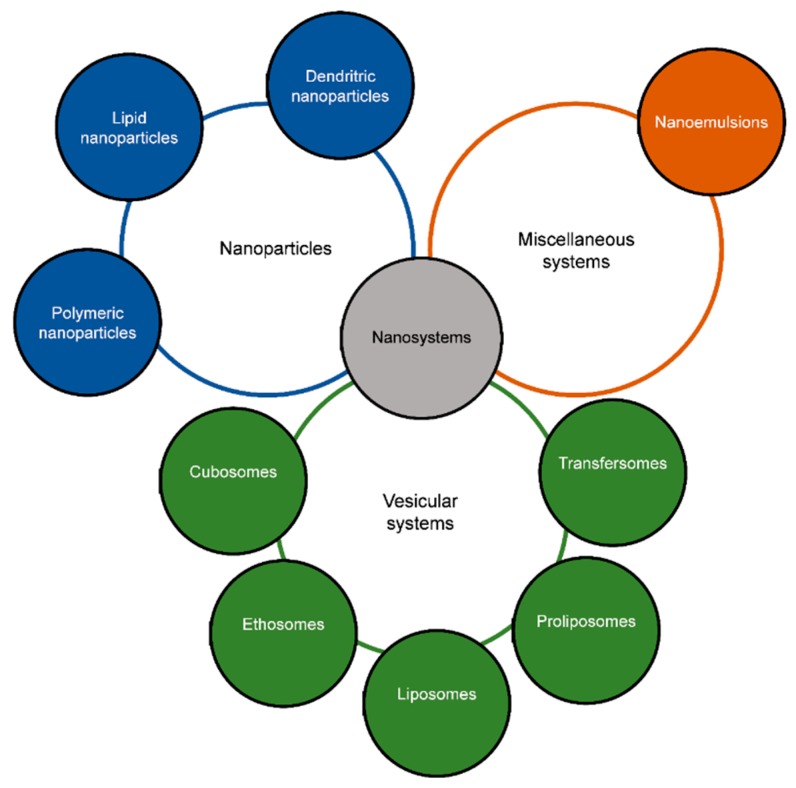
Types of nanoparticles commonly used for topical delivery of drugs.

Nanoparticles (NPs) stand for materials with dimensions less than 100 nm [46,47], and have been proposed for the topical delivery of drugs aimed at treating skin diseases [32]. NPs may contribute to reduce adverse effects of classical drugs (e.g., topical corticosteroids), as the former show improved safety profile as less drug is needed due to the site specific delivery. NPs have also been proposed as a preferential approach to poor drug solubility and limited skin permeability, thereby increasing skin bioavailability [48,49]. Several types of nanoparticles have been proposed for the topical delivery of a set of different drugs useful in AD, e.g., antibiotics and corticosteroids [7,8]. A schematic representation of types of nanoparticles used in topical delivery of drugs is shown in Figure 1.

NPs offer opportunities to improving drug penetration through the SC, increase the retention time of drugs, and design better profiles of drug release to reach therapeutic goals. NP-based formulations may, therefore, be an improved approach over traditional drug formulations. One of the most relevant advantages of NPs is the potential reduction of serious adverse effects related to poor patient compliance and, thus, reduced therapeutic outcomes [3,50]. Among the different NPs, those composed of lipid materials have shown superior advantages attributed to the compatibility with the lipid skin composition [48,51,52,53,54].

### 7.1. Vesicular Systems

#### 7.1.1. Liposomes

Liposomes are formed by ordinary phospholipids found in living systems that are disposed in concentric bilayers. This display generates a hydrophilic environment in the core of the vesicles and a lipophilic environment between the layers of phospholipids. Cholesterol is employed in the manufacturing of liposomes to avoid phospholipid aggregation and to allow membrane fluidity. Liposomes are versatile structures showing interesting characteristics as biocompatibility, increased ability to penetrate and permeate across the epidermis (feature derived from the lipid-similarity to this biological structure), and the possibility to encapsulate both lipophilic and hydrophilic molecules. These properties make liposomes a very reliable source of drug carriers [3,4,32].

#### 7.1.2. Transfersomes

Transfersomes show many structural similarities with liposomes but exhibit higher flexibility in penetrating through the SC via intracellular and/or intercellular routes. They can also pass through pores smaller than their diameter because of their flexibility, a feature attributed to their amphipathic assembly containing a surfactant [55].

#### 7.1.3. Ethosomes

Ethosomes are also structurally similar to liposomes. Due to their higher flexibility they are also called elastic vesicles. Ethosomes are classified into three distinct categories: classical ethosomes, binary ethosomes and transethosomes. The composition of classical ethosomes is based on phospholipids, highly concentrated ethanol, and water. Binary ethosomes have an identical composition to classical ethosomes but replacing the alcohol by propylene glycol or isopropyl alcohol. Transethosomes are also identical to classical ethosomes with the exception of the additional surfactant that allows flexibility of vesicles and penetration across pores smaller than their sizes [56,57,58]. Ethosomes-derived formulations together with transfersomes can cross pores smaller than their diameter due to the high fluidity of the lipidic membrane. Ethosomes were experimentally shown to efficiently deliver targeted drugs in a higher extent than liposomes, and other solutions based on ethanol and water [3,59,60].

#### 7.1.4. Proliposomes

Proliposomes are vesicular carriers forming liposomes after entropy-driven reconfiguration. They show a trend towards aggregation due to the lack of water molecules, which contribute to protein stability. Once the shell of the vesicular carrier is hydrated the drug is released with parallel rise of skin permeation. As these structures are easily scaled-up to meet industrial processing requirements, they have become an interesting method to deliver therapeutic molecules [61].

#### 7.1.5. Cubosomes

Cubosomes were developed to encapsulate peptide- and protein-derivative drugs given their ability to prevent enzymatic modifications. They are generated by emulsifying a lipid phase with cubic geometry in water yielding NPs in liquid state with crystalline properties. Given their structure, lipophilic drugs can be encapsulated by incorporation in the lipid bilayers, and hydrophilic drugs can be encapsulated within the aqueous channels in the structure of the vesicles. Amphipathic drugs, on the other hand, are placed between the lipid bilayer and the water phase. These vesicles have optimized parameters as low viscosity, increased stability to elevated temperatures, and a higher surface area. The drug-releasing mechanism govern the percutaneous absorption of drugs [3,62,63,64].

### 7.2. Nanoparticles

#### 7.2.1. Lipid Nanoparticles

Lipid nanoparticles refer to solid lipid nanoparticles (SLN) and nanostructured lipid carriers (NLC), both among the range of NPs of interest for skin drug delivery [48,49,65,66]. SLN are mainly composed of solid lipids. Whereas, NLC are composed of a blend of solid and liquid lipids; both SLN and NLC matrices remain solid at body and room temperatures. Both lipophilic and hydrophilic drugs are prone to be encapsulated in SLN, while, NLC are specifically tailored to load lipophilic drugs. SLN and NLC improve drug permeation through the SC and retain them both in epidermis and dermis. SLN and NLC can also re-establish the health of the skin as they reduce the transepidermal water loss, a property linked to the adhesiveness of the particles and to their minor size, yielding occlusion of the skin and, therefore, preventing water evaporation [37,67].

#### 7.2.2. Polymeric and Polysaccharide Nanoparticles

Polymeric nanoparticles commonly employ polylactic acid (PLA), poly(ε-caprolactone) or poly(lactic-co-glycolic acid) (PLGA) [68,69,70,71,72,73], whereas polysaccharide nanoparticles are based on, e.g., chitosan [74], alginate [75,76], all non-toxic, biocompatible, and biodegradable polymers.

#### 7.2.3. Dendritic Nanoparticles

Dendritic NPs allow the conjugation of a diversity of molecules in their shells. Amongst those characteristics is the flexibility permitted by the macromolecules constituting these systems, the small polydispersity index, as well as the hyperbranched lattice with a regular stride [77,78].

### 7.3. Nanoemulsions

Nanoemulsions are isotropic binary systems, composed of two immiscible liquids, forming oil droplets of nanometer size dispersed in aqueous phase and stabilized by at least one surfactant. Unlike microemulsions, nanoemulsions are thermodynamically unstable, showing higher capacity to load lipophilic drugs. In biological systems, they have the ability to penetrate through healthy or diseased skin. Nanoemulsions also have the potential to increase the bioavailability of drugs. This phenomenon is derived from their positive charge, which is attracted by the negatively charged corneocytes, present in the SC leading to enhanced percutaneous drug absorption [51,53,79,80,81].

## 8. Production of Nanoparticles Delivery Systems

**Figure 2:**
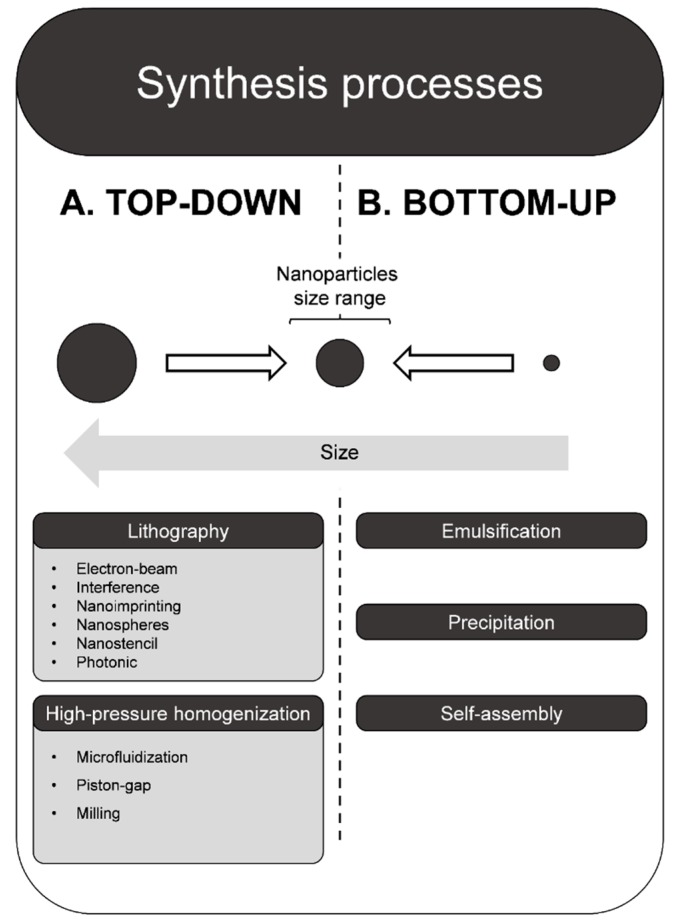
Top-down and bottom-up strategies for the production of nanoparticles.

For the production of nanoparticles, two strategies are described, namely top-down or bottom-up (Figure 2).

### 8.1. Top-Down

Top-down approaches are based on the reduction of macroparticles until these reach the nanometric scale. To accomplish so, a variety of methods are available as lithography, which includes derived methods, such as nanoimprinting, electron-beam, nanostencil, photonic, interference, nanospheres, high-pressure homogenization, including homogenization (e.g., microfluidization, piston-gap) and milling. One of the main advantages of top-down approaches is the preservation of the crystalline structure of the NPs. Although, this is the main common outcome, the development of an amorphous structure does often occur throughout the process of milling. To promote recrystallization, a solvent is frequently used to obtain pure drug NPs. Regarding the method of microfluidization, a critical factor to ensuring the reduction of the particle size is the presence of a jet of air, which acts as a milling system by promoting the impact between particles. A disadvantage of top-down approaches is the need for high input of energy, which is often expensive [82,83].

### 8.2. Bottom-Up

Whilst top-down approaches are based on the reduction of materials to achieve a proper formulation, bottom-up methodologies start with smaller building-blocks and assemble them in bigger structures. Among these, self-assembly, precipitation, or emulsification of starting-materials are commonly applied. From a practical perspective, this is reached by setting a drug or a mix of drugs into a solution. Then, using procedures that precipitate or crystalize the drugs in solution, NPs are built up. The last step consists of evaporating the solvent to yield the desired constructs. A strong advantage of these methodologies is the minor amount of energy needed along with small economical expenditures. Nonetheless, the techniques are prone to yield both crystalline and amorphous structures of NPs, and associated irregularities in parameters, such as shape, size of the NPs, and the respective polydispersity index [82,83].

## 9. Nanoparticles and Skin Penetration

The penetration of the NPs applied topically is done by at least one of three routes, i.e., intracellular route, intercellular route or through hair follicles and sweat glands. Critical governing parameters the NPs permeability through the skin are their size, the type of surfactant surrounding the particles and their surface electrical charge (i.e., zeta potential). If NPs have a mean diameter not superior to 4 nm, they can easily penetrate or permeate through the intact skin; if the diameter is between 4 and 20 nm, NPs can permeate via damaged or intact skin; if the diameter of the NPs is under 45 nm they can be permeated or penetrate insalubrious skin. For sizes greater than 45 nm, NPs, susceptible to be accumulated or translocated in, or through, skin appendices, proved difficult in penetrating and permeating the skin [84].

The loading of drugs into NPs can potentially improve their physicochemical stability, their bioavailability, and reduce their adverse side effects [2]. NPs can also reduce the overall tissue capacity of clearance, and thereby, increase the drug concentration in the diseased tissue. Due to the controlled release profile of the entrapped drug and the high surface area, NPs are an interesting approach for site-specific drug delivery to the skin [29,30,47]. Negatively charged NPs can interact with positively charged keratinocytes, thereby, enhancing the NPs retention onto the skin and extended drug release.

## 10. Nanoparticles for Atopic Dermatitis

Chitosan NPs, containing betamethasone valerate, were produced and coated with hyaluronic acid to improve the pharmacological effects of the drug and the capacity for targeted delivery [85]. NPs displayed a Fickian diffusion release profile in simulated skin, with enhanced drug permeation in comparison to the non-loaded drug. Coating the particles with hyaluronic acid better improved drug retention in the epidermis and the dermis.

Siddique et al. loaded hydrocortisone and hydroxytyrosol—a glucocorticoid for topical application, and an antioxidant molecule, respectively, in chitosan NPs, which were then formulated in an aqueous cream [86]. The authors reported no systemic effects of the drugs nor toxicity, with enhanced safety and tolerability of the developed cream. Chitosan NPs loading hydrocortisone showed their capacity to reduce the erythema, diminish the thickness of the affected skin areas, and control the transepidermal water loss [87]. The proposed NPs are suitable in delivering glucocorticoid-related drugs, in order to decrease the fibrotic and inflammatory patterns, and to improve the elasticity of the connective tissues in AD patients.

Transfersomes loading tacrolimus were compared with liposomes containing the same drug and with the ointment marketed product (Protopic^®^). Transfersomes achieved higher retention of tacrolimus than the other two [88].

Chitosan nanoparticles have been proposed for the delivery of nicotinamide and tacrolimus for the treatment of AD [89], and their in vitro and in vivo permeation compared to a commercial ointment (Protopic). The authors reported that the enhanced permeation effect of NPs through, and into, the skin, contributed to increased amounts of tacrolimus in the skin. This approach has been proposed as adjuvant therapy and moderate anti–AD effects.

An in vivo study in mice has described the topical application of guar gum NPs, which were shown to reduce the cellular infiltration and epidermal thickness in oxazolone-induced AD [1]. The NPs used in this study were derived from *Cyamopsis tetragonoloba*, which contains galactomannan, known to increase the cellular uptake of macrophages mediated by their over-expressed mannose receptors.

NPs made of chitosan were loaded with tacrolimus and further covered with hyaluronic acid [90]. The results from in vitro studies demonstrated that tacrolimus was kept both in epidermal and dermal layers—proof of successful targeting—decreasing the overall intensity of erythema and the transepidermal water loss. Particles covered with hyaluronic acid showed other promising effects in the skin, as anti-dermatitis, which point to a possible successful system in targeting immunomodulatory drugs.

Lipid NPs, loaded with tacrolimus, improved penetration across the skin, reaching the more profound layers (with resident dendritic cells) [91]. The comparison between tacrolimus-loaded lipid NPs and the ointment Protopic^®^ revealed that the former had boosted bioavailability. Systemic distribution of the drug was not observable, which constitutes a favourable factor.

A system consisting of flexible vesicles loaded with levocetirizine was assessed on its safety and dermatologic ability to pass through the skin. The results confirmed the criteria of skin safety and demonstrated that this system was able to penetrate/permeate across the skin and had higher retention capacity in comparison to liposomes, due to the flexible features of the vesicles [92].

**Figure 3:**
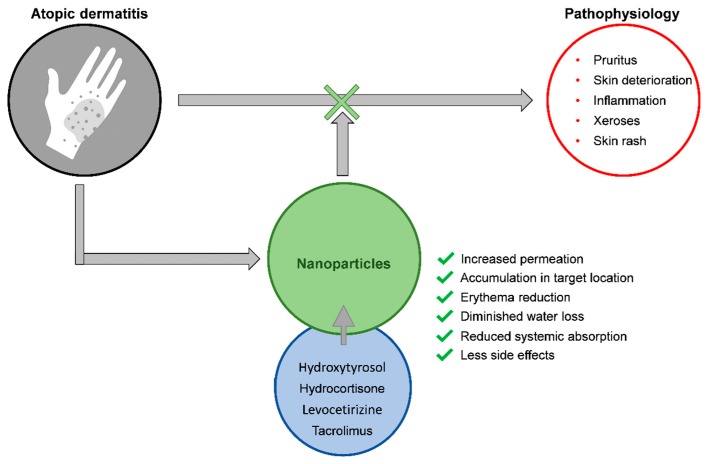
Nanoparticles as potential strategies for the treatment of atopic dermatitis.

A study also employing the antihistamine levocetirizine, but formulated in elastic vesicles and topically applied, showed an increased flexibility of the membranes of these vesicles, a critical feature allowing the vesicles to pass through pores in the human body with sizes smaller than their dimensions. The increased permeability of the drug, along with reduced erythema intensity and itching, were also reported [93]. A schematic representation of nanoparticles used as potential strategies for the treatment of atopic dermatitis is shown in Figure 3.

## 11. Toxicological Concerns and Legal Issues

The main concern when using NPs as drug delivery systems is their cytotoxicity profile. NPs interact with cells at different levels and may also induce an immunological response. Neutral or negatively charged NPs are better tolerated than their positively charged counterparts [94]. Negatively charged NPs of mean diameter below 200 nm showed a dramatic reduction in the discharge of pro-inflammatory factors when concomitantly administered with 1-fluoro-2,4-dinitrobenzene (DNFB), which is a Th1-cell sensitizer [95]. This mechanistic is explained by the inhibition of the degranulation process of mast cells with a later enrolment of other types of immune cells. Other studies showed typical immune responses when free DNFB was applied. Keratinocytes demonstrated an enhanced production of cytokines as IL-1β and IL-18, responsible for triggering and expanding the activation and degranulation of mast cells, with further release of histamine [94,96].

The overall toxicity triggered by NPs can be assessed at distinct levels, namely, at molecular, cellular, tissue, and organ level [54], as drug pathway differ if it is loaded within NPs or if it is free. The smaller the NPs, the higher the ability to penetrate across the skin, allowing improved access to systemic circulation, and therefore, to several organs and body tissues. The decrease of the mean particle size may increase their reactivity with consequent harmful effects in vivo. The phenomenon of protein aggregation surrounding the NPs surface is commonly observed [97]. NPs may trigger cell toxicity through the generation of reactive oxygen species (ROS) by Fenton’s reaction. ROS are damaging and lead to the loss of lysosome membrane integrity with later release of enzymatic hydrolytic machinery, iron cations, and protons. These latter lead to mitochondrial dysfunction, protein aggregation, and increased cellular oxidative stress [98,99]. Toxicological assays are, therefore, of utmost importance in forecasting the likely toxicity of given NPs [98].

The legislation governing nanotechnology-derived medicines does not need to have extremely detailed guidelines. Nevertheless, it is vital that the medicines are in conformity with the current rules of safety to avoid any toxicological risks.

## 12. Conclusions

Overall, AD is primarily characterized by its chronic pattern along with important modifications in the quality of life of patients and caretakers. The first-line treatments aim to reduce the pruritus, associated inflammation, and to rebuild the integrity of the skin using drugs as topical corticosteroids and/or topical calcineurin inhibitors. Despite this, chronic treatment using these compounds, and in particular, topical corticosteroids, leads to skin shrinkage due to the inhibition of collagen synthesis. In children, there is an associated risk of systemic distribution of these drugs, which is a result of their barely developed skin, and subsequent harmful adverse side effects. Topical treatments with nanoparticles avoid the absorption of considerable amounts of drug due to the limited capacity of the former to penetrate the stratum corneum. Since AD modifies skin homeostasis, novel treatments, focusing on the restoration of the normal function of the skin, intended to regulate and stop the associated inflammation, are urgently required. In the last years, the daily usage of NPs grew massively, mostly due to their remarkable properties. Among these uses include; the ability to be targeted to a tissue or cells; the possibility to achieve an easier control of the drug release profile and the ability to penetrate/permeate the skin, allowing the NPs to be in contact with the most profound layers of it, decrease in skin irritation; and the overall adverse effects of the drug, thereby, improving patient acquiescence to therapeutics. The challenge concerns the therapeutic agents and medicines available. Approaches using NPs-derived systems in the therapy of AD are being exploited. There are many advantages as the effectiveness of the medicine and its lower cost, the reduced administered doses (as NPs are very efficient in the targeted delivery which allows the medicine to be administered with longer intervals) increasing patients’ compliance, and reducing the overall toxicity as the drug is retained in the skin and is targeted. Novel nano-engineered systems are currently being tested in AD models, and are promising for the future treatment of this disease. NPs may be capable in reducing the adverse side effects of currently available treatments of AD. Despite this, it is of utmost importance to guarantee the safety and efficacy of NPs, allowing the generation of potentially marketable products.

## Figures and Tables

**Table 1:** Overview of the current therapeutic approaches in Atopic Dermatitis (AD).

Antihistamines	Diphenhydramine Hydroxyzine
Anti-microbials	Systemic antibiotics (e.g., cyclosporine) Topical antibiotics (e.g., gentamicin, fusidic acid, mupirocine)
Corticosteroids	Systemic corticosteroids (e.g., prednisolone) Topical corticosteroids (e.g., hydrocortisone, fluticasone, betamethasone)
Education	Advantages of treatment compliance Application of topical therapy Nature of the disease
Phototherapy	UVB light UVA light
Skincare	Moisturizers Daily skincare Emollients Regular bathing
Systemic immunosuppressant drugs	Azathioprine Ciclosporin Methotrexate Mycophenolate mofetil
Topical calcineurin inhibitors	Pimecrolimus Tacrolimus
